# Sex-specific echocardiographic remodeling and prognostic value of left ventricular sphericity, pressure–dimension index, and myocardial work after durable LVAD implantation

**DOI:** 10.3389/fcvm.2026.1815815

**Published:** 2026-06-23

**Authors:** Mahmoud Jaber, Ahmed F. A. Mohammed, Yusuf Shieba, Ajay Moza, Alish Kolashov, Rashad Zayat

**Affiliations:** 1 Department of Cardiac Surgery, Medical Faculty, RWTH Aachen University, Aachen, Germany; 2 Department of Cardiothoracic Surgery, Faculty of Medicine, Qena University, Qena, Egypt; 3 Department of Cardiothoracic Surgery, Heart Centre Trier, Barmherzigen Brueder Hospital, Trier, Germany

**Keywords:** cardiac remodeling, left ventricular assist device, myocardial work, right heart failure, sex differences, sphericity index

## Abstract

**Background:**

Sex-related differences in outcomes after left ventricular assist device (LVAD) implantation are recognized, yet the structural mechanisms remain incompletely defined. We evaluated sex-specific remodeling patterns and the prognostic value of ventricular geometry and non-invasive myocardial work (MW).

**Methods:**

We performed a retrospective analysis of 103 adults (85 men, 18 women) undergoing durable LVAD implantation. Primary endpoints were changes in left ventricular sphericity index (LVSI) and RV pressure–dimension index (PDI) from baseline to 6 months. Adjusted models (ANCOVA with robust standard errors) and quantile regression were used to assess sex effects, with robustness examined by a 1:2 propensity score matching analysis (matching on age, BMI, clinical profile, and device). Predictors of mortality and postoperative right heart failure (RHF) were assessed using Cox and logistic regression models, respectively.

**Results:**

Baseline geometry and myocardial work were similar between sexes. Post-implantation, men exhibited adverse sphericalization (median ΔLVSI +0.045, *p* = 0.001) and a significant decline in PDI (median Δ −1.475, *p* < 0.001). In contrast, women demonstrated a pattern of “geometric resistance” [defined descriptively as stable left ventricular sphericity index (ΔLVSI *p* = 0.766) and a non-significant change in RV pressure-dimension index (median ΔPDI −0.922, *p* = 0.130)], while maintaining higher absolute PDI at 6 months (*p* = 0.041). While statistical significance was attenuated after 1:2 propensity-score matching, the matched-cohort estimates remained directionally consistent but imprecise: adjusted 6-month LVSI difference −0.060 (95% CI −0.189 to 0.069; *p* = 0.342), adjusted mean PDI difference +0.276 (95% CI −0.589 to 1.140; *p* = 0.510), and adjusted median PDI difference +0.828 (95% CI −0.150 to 1.806; *p* = 0.095). Preoperative LVSI was independently associated with mortality (HR 1.47 per SD, *p* = 0.034). Furthermore, preoperative global work index (GWI) was a robust independent predictor of both mortality (HR 0.14 per SD, *p* < 0.001) and postoperative right heart failure (OR 0.41 per SD, *p* = 0.022), with larger effect sizes than sex in adjusted models.

**Conclusion:**

In this exploratory cohort, women showed preserved LV geometry and PDI after LVAD implantation, but findings require cautious interpretation due to small sample size and attenuation after matching. LVSI and myocardial work may support future risk stratification studies.

## 1 Introduction

Durable left ventricular assist devices (LVADs) have become an established therapy for advanced heart failure as bridge-to-transplantation, bridge-to-decision, or destination therapy, with contemporary devices improving survival and reducing major device-related complications compared with earlier generations (1, 2). Despite these advances, postoperative morbidity remains substantial, and accurate preoperative risk stratification is still a key clinical need (1).

Right heart failure (RHF) after LVAD implantation is among the most impactful complications, associated with prolonged intensive care, need for temporary RV support, renal failure, and worse short- and long-term outcomes (3, 4). Echocardiography is central to perioperative assessment because it offers a non-invasive evaluation of biventricular size, geometry, loading/unloading patterns, and RV performance, and guideline-based multimodality imaging has been emphasized for optimized LVAD patient management (5). In addition, sex-related differences in patient characteristics and outcomes after LVAD have been repeatedly reported, motivating analyses that disentangle “biological” sex effects from differences in baseline risk profile and illness severity (6, 7).

Beyond conventional indices, LV geometry-based parameters may capture clinically relevant remodeling (8). The LV sphericity index (LVSI) and the pressure–dimension index (PDI) have been proposed as additional echocardiographic metrics that reflect ventricular shape and pressure–size interaction. Prior work has suggested these indices hold prognostic value for postoperative RV failure and longer-term outcomes in LVAD cohorts (9, 10), as well as in broader heart failure populations (11). However, how LVSI and PDI evolve during early reverse remodeling after LVAD implantation—and whether their trajectories differ by sex—remains incompletely characterized.

Non-invasive myocardial work derived from pressure–strain analysis integrates myocardial deformation with afterload and may provide incremental information beyond ejection fraction or strain alone (12–14). In LVAD candidates, preoperative global myocardial work indices have been reported to improve the prediction of mortality and RHF beyond established clinical variables (15). Moreover, pressure-strain-derived metrics appear to reflect LV stroke work across a wide range of LVAD support levels, supporting their physiologic relevance in mechanically unloaded ventricles (16).

Therefore, the aims of this study were (i) to compare sex-specific changes in LV geometry (LVSI and PDI) from pre-implantation to 6 months after LVAD implantation and (ii) to evaluate associations of preoperative geometry and myocardial work parameters with clinically relevant outcomes, including survival and RHF.

## 2 Methods

### 2.1 Study design and population

**Figure 1:**
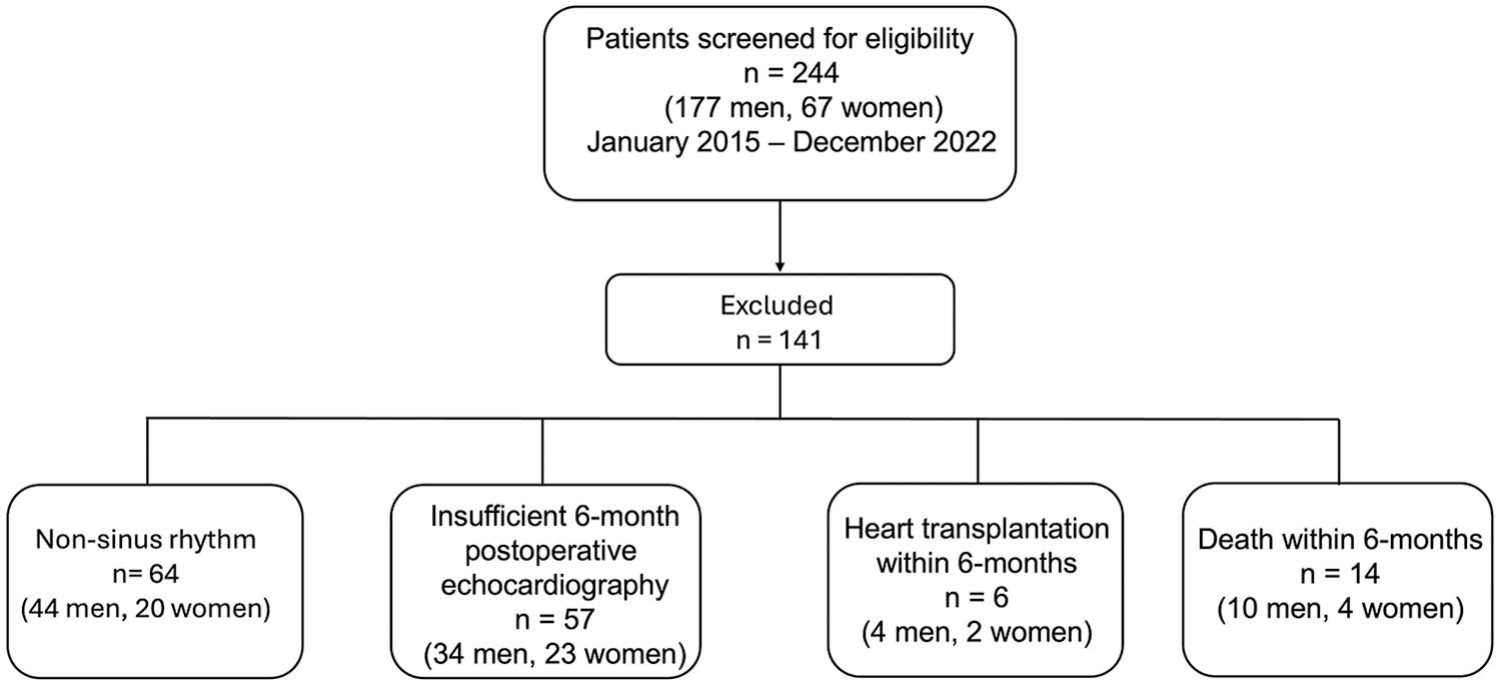
STROBE-style patient flow diagram. Flow diagram showing screening, exclusion criteria, and final cohort selection. Of 244 LVAD recipients screened between January 2015 and December 2022, 141 were excluded, resulting in a final study cohort of 103 patients: 85 men and 18 women.

This study was a retrospective analysis of end-stage heart failure patients who were candidates for durable left ventricular assist device (LVAD) implantation at our department between January 2015 and December 2022. Of 244 consecutive patients who underwent durable LVAD implantation between January 2015 and December 2022, 141 were excluded for the following reasons: non-sinus rhythm or frequent extrasystoles precluding reliable speckle-tracking myocardial work analysis (*n* = 64; 44 men, 20 women), insufficient high-quality paired pre- and 6-month postoperative echocardiography (*n* = 57; 34 men, 23 women), heart transplantation before the 6-month window (*n* = 6; 4 men, 2 women), or death before the 6-month window (*n* = 14; 10 men, 4 women). Patient selection is illustrated in Figure 1 (STROBE flow diagram).

Inclusion criteria were: age ≥18 years; end-stage heart failure with severely reduced ejection fraction (EF < 25%); sinus rhythm without notable extrasystoles; availability of preoperative and 6-month follow-up transthoracic echocardiography (TTE) enabling measurement of the LVSI (17, 18) and PDI (9); for non-invasive myocardial work (MW) analysis, additional requirements were adequate image quality for speckle-tracking and availability of a contemporaneous brachial blood pressure measurement for pressure calibration, consistent with established MW methodology; and heart failure etiology of ischemic heart disease (ICM) or idiopathic dilated cardiomyopathy (DCM). Follow-up was administratively censored on December 31, 2024, with a median follow-up of 44 months (IQR 8–56). The study was approved by the Research Ethics Committee of RWTH University Aachen (EK 151/09), which waived the requirement for informed consent due to the retrospective study design.

### 2.2 Data collection and risk stratification

Demographics, clinical progression, and perioperative surgical and anesthetic protocols were extracted from our institutional electronic database. Laboratory data and the occurrence of adverse events, including thromboembolic events, hemorrhage, right heart failure (RHF), death, and acute kidney injury (AKI), were gathered. Adverse events were defined according to the Interagency Registry for Mechanically Assisted Circulatory Support (INTERMACS) 2021 guidelines (19). INTERMACS level was calculated for all patients.

### 2.3 Transthoracic echocardiography acquisition and analysis

TTE examinations were performed preoperatively and at 6 months post-implantation as part of routine follow-up, using commercially available ultrasound machines (GE Vivid E90, GE Vingmed Ultrasound, Horten, Norway) in accordance with contemporary guideline recommendations for imaging in patients receiving mechanical circulatory support (5, 17). Standard 2D and Doppler datasets (three cardiac cycles where feasible) were digitally stored and analyzed offline using vendor software (EchoPAC, GE Vingmed). Left ventricular ejection fraction (LVEF) was measured using the modified biplane Simpson method. Right ventricular systolic function indices included tricuspid annular plane systolic excursion (TAPSE) and tissue-Doppler tricuspid annular systolic velocity (TASV). Systolic pulmonary artery pressure (SPAP) was estimated from tricuspid regurgitation velocity with standard assumptions for right atrial pressure when required.

LVSI was calculated in the apical four-chamber view at end-diastole as the ratio of the LV basal short-axis diameter (radial/basal dimension) to the LV longitudinal length (base-to-apex) (17, 18). Right ventricular PDI was calculated using the following equation: SPAP/right ventricular minor diameter (RVMD)^2^, where RVMD is measured immediately above the tricuspid valve at end-diastole (9).

MW indices were derived from pressure–strain loop analysis by combining global longitudinal strain (GLS) obtained by 2D speckle tracking with an estimated LV pressure curve scaled to brachial cuff systolic pressure (12, 13). Global MW parameters included: global work index (GWI), global constructive work (GCW), global wasted work (GWW), and global work efficiency (GWE).

### 2.4 Statistical analysis

Continuous variables are reported as median (interquartile range [IQR], 25th–75th percentile) and categorical variables as *n* (%). Between-sex comparisons used the Mann–Whitney *U* test for continuous variables and *χ*^2^ or Fisher's exact test for categorical variables, as appropriate.

The primary remodeling endpoints were sex-related differences in LVSI and PDI from baseline to 6 months after LVAD implantation. Within-group changes were assessed using the paired Wilcoxon signed-rank test, and between-sex differences were assessed using the change score (Δ = 6 months − baseline). To adjust for baseline differences and regression to the mean, we additionally used ANCOVA-style models with the 6-month value as outcome (6-month value ∼ sex + baseline value + age + BMI + INTERMACS level + device type), applying robust standard errors. Because PDI was right-skewed, quantile (median) regression was prespecified as a complementary approach to robust mean-based linear models.

To enhance robustness in a cohort with fewer female patients, a prespecified sensitivity analysis used propensity score matching (1 female:2 males) with nearest-neighbor matching based on age, BMI, INTERMACS level, and device type. Covariate balance was evaluated using standardized mean differences (SMD) and visualized using a Love plot; absolute SMD < 0.10 was considered acceptable for matching covariates.

For secondary outcomes, associations of preoperative LVSI, PDI, and myocardial work indices with mortality were assessed using Cox proportional hazards models (hazard ratio [HR] and 95% confidence interval [CI]). Associations with postoperative right heart failure (RHF) were assessed using logistic regression (odds ratio [OR] and 95% CI). Continuous predictors were scaled per 1 standard deviation (SD) to facilitate comparability across measures. An exploratory outcome sensitivity analysis was performed in the broader available LVAD cohort with available survival time, vital status, and postoperative RHF status, including patients initially excluded from the primary echocardiographic analysis. Sex-specific survival was assessed using Kaplan–Meier analysis, log-rank testing, and Cox proportional hazards regression. Postoperative RHF was assessed using Fisher's exact test and logistic regression. Adjusted models included sex, age, BMI, INTERMACS level, and device type. Two-sided *p*-values <0.05 were considered statistically significant. Analyses were performed using R (version 4.4.2; R Foundation for Statistical Computing, Vienna, Austria) on macOS.

## 3 Results

### 3.1 Patient cohort and baseline characteristics

A total of 103 patients were included (85 men, 18 women). The final cohort of 103 patients with paired pre- and 6-month echocardiography was derived from 244 screened patients (see Figure 1 for STROBE flow diagram). Overall mortality was 28.2% (29/103) and postoperative right heart failure (RHF) occurred in 23.3% (24/103). Baseline demographic, clinical, and echocardiographic characteristics were broadly comparable between sexes (Table 1). There were no significant differences in age (median 62.0 vs. 62.5 years, *p* = 0.838), body mass index (BMI; *p* = 0.636), body surface area (BSA; *p* = 0.174), renal function (creatinine; *p* = 0.980), or baseline left ventricular ejection fraction (LVEF; *p* = 0.788). Baseline LV geometry and PDI were similar between sexes (baseline LVSI *p* = 0.125; baseline PDI *p* = 0.346). Myocardial work indices were also comparable (e.g., GWI *p* = 0.241), with a numerical trend toward lower GWE in women (*p* = 0.084). Device type distribution was similar (HM3: 47/85 [55.3%] in men vs. 10/18 [55.6%] in women; HMII: 38/85 [44.7%] vs. 8/18 [44.4%]; *p* = 1.000; Table 1).

**Table 1:** Baseline characteristics, preoperative echocardiography, and outcomes by sex (full cohort).

Variable	Male (*n* = 85)	Female (*n* = 18)	*p*
Demographics and clinical variables			
Age, years	62 (57–67)	62.5 (55.8–67.8)	0.838
Body mass index (BMI), kg/m^2^	26.6 (23.2–30.3)	27.3 (25.1–29.7)	0.636
Body surface area (BSA), m^2^	2 (1.83–2.16)	1.93 (1.84–2.08)	0.174
Serum creatinine, mg/dL	1.1 (0.975–1.3)	1.02 (0.927–1.56)	0.980
Left ventricular ejection fraction (LVEF), %	23 (19–27)	23 (19.7–26.9)	0.788
Preoperative echocardiography and myocardial work			
Preoperative left ventricular sphericity index (LVSI)	0.663 (0.587–0.791)	0.742 (0.616–0.825)	0.125
Preoperative RV pressure–dimension index (PDI), mmHg/cm^2^	3.04 (2.15–4.34)	2.52 (2.07–3.18)	0.346
Global work index (GWI), mmHg%	405 (205–617)	219 (167–452)	0.241
Global constructive work (GCW), mmHg%	557 (290–782)	483 (302–690)	0.380
Global work efficiency (GWE), %	67 (56–78)	63.5 (56.2–66)	0.084
Global wasted work (GWW), mmHg%	231 (159–347)	282 (161–345)	0.543
Global longitudinal strain (GLS), %	−5 (−7–−4)	−5 (−7–−1.25)	0.812
Tricuspid annular plane systolic excursion (TAPSE), mm	16 (12.2–20)	16.5 (12.2–19.5)	0.731
Estimated systolic pulmonary artery pressure (sPAP), mmHg	32.6 (24.7–41.2)	26.6 (19.6–37.5)	0.203
Device type and comorbidities			
Device type, *n* (%)	—	—	1.000
HeartMate 3 (HM3)	47 (55.3%)	10 (55.6%)	
HeartMate II (HMII)	38 (44.7%)	8 (44.4%)	
Ischemic cardiomyopathy (ICM)	59 (69.4%)	10 (55.6%)	0.279
Dilated cardiomyopathy (DCM)	24 (28.2%)	5 (27.8%)	1.000
Diabetes mellitus	15 (17.6%)	2 (11.1%)	0.730
Peripheral artery disease (PAD)	16 (18.8%)	4 (22.2%)	0.747
Arterial hypertension	64 (75.3%)	14 (77.8%)	1.000
Chronic obstructive pulmonary disease (COPD)	21 (24.7%)	8 (44.4%)	0.082
Pulmonary hypertension	50 (58.8%)	11 (61.1%)	0.790
Prior cardiac surgery	13 (15.3%)	2 (11.1%)	1.000
Prior PCI/stenting	37 (43.5%)	6 (33.3%)	0.597
Concomitant procedures			
Isolated LVAD implantation	43 (50.6%)	13 (72.2%)	0.121
LVAD + CABG	22 (25.9%)	3 (16.7%)	0.550
LVAD + TVR	15 (17.6%)	2 (11.1%)	0.730
LVAD + AVR	5 (5.9%)	0 (0.0%)	0.583
Outcomes			
All-cause mortality during follow-up	24 (28.2%)	5 (27.8%)	1.000
Postoperative right heart failure (RHF)	20 (23.5%)	4 (22.2%)	1.000
Postoperative RVAD implantation	5 (5.9%)	0 (0.0%)	0.584

Values are median (interquartile range [IQR], 25th–75th percentile) or *n* (%). Between-group comparisons were performed using the Mann–Whitney *U* test for continuous variables and *χ*^2^ test or Fisher's exact test for categorical variables, as appropriate. *P*-values are two-sided. AVR, aortic valve replacement; BSA, body surface area; CABG, coronary artery bypass grafting; COPD, chronic obstructive pulmonary disease; DCM, dilated cardiomyopathy; GCW, global constructive work; GLS, global longitudinal strain; GWE, global work efficiency; GWI, global work index; GWW, global wasted work; HM3, HeartMate 3; HMII, HeartMate II; ICM, ischemic cardiomyopathy; IQR, interquartile range; LVAD, left ventricular assist device; LVEF, left ventricular ejection fraction; LVSI, left ventricular sphericity index; PAD, peripheral artery disease; PDI, right ventricular pressure–dimension index; PCI, percutaneous coronary intervention; RHF, right heart failure; RVAD, right ventricular assist device; RV, right ventricle; sPAP, systolic pulmonary artery pressure; TAPSE, tricuspid annular plane systolic excursion; TVR, tricuspid valve repair.

### 3.2 Sex-specific remodeling from baseline to 6 months

**Figure 2:**
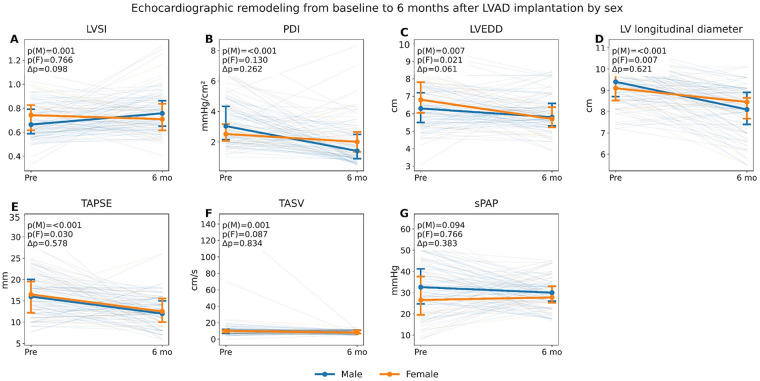
Sex-stratified echocardiographic remodeling from baseline to 6 months after LVAD implantation. (**A)** LV sphericity index (LVSI), **(B)** right ventricular pressure–dimension index (PDI), **(C)** LV end-diastolic diameter (LVEDD), **(D)** LV longitudinal diameter, **(E)** tricuspid annular plane systolic excursion (TAPSE), **(F)** tricuspid annular systolic velocity (TASV), and **(G)** systolic pulmonary artery pressure (sPAP). Thin lines represent paired measurements for individual patients. Circles with error bars indicate median and interquartile range (IQR; 25th–75th percentile) at each time point. *P*-values shown within each panel denote: p(M), within-male comparison of baseline vs. 6 months (paired Wilcoxon signed-rank test); p(F), within-female comparison of baseline vs. 6 months (paired Wilcoxon signed-rank test); Δp, between-sex comparison of the change score (Δ = 6 months−baseline; Mann–Whitney *U* test).

Sex-stratified changes from baseline to 6 months are summarized in Table 2 and visualized in the multi-panel paired remodeling figure (Figures 2A–G).

**Table 2:** Echocardiographic changes from baseline to 6 months.

Parameter	Male pre	Male 6m	Male Δ	Male paired p	Female pre	Female 6m	Female Δ	Female paired *p*	Between-sex *p* (Δ)
LV Sphericity index	0.663 (0.587–0.791)	0.757 (0.65–0.862)	0.0445 (−0.0282 to 0.18)	0.001	0.742 (0.616–0.825)	0.708 (0.615–0.837)	−0.0567 (−0.218 to 0.225)	0.766	0.098
RV Pressure–dimension index	3.04 (2.15–4.34)	1.41 (0.9–2.5)	−1.48 (−2.75 to −0.417)	<0.001	2.52 (2.07–3.18)	2.01 (1.35–2.66)	−0.922 (−1.78 to 0.237)	0.130	0.262
LVEDD (cm)	6.3 (5.5–7.2)	5.8 (5.3–6.59)	−0.43 (−1.1 to 0.3)	0.007	6.8 (6.05–7.81)	5.7 (5.22–6.38)	−1.05 (−2.33 to −0.425)	0.021	0.061
LV longitudinal diameter	9.4 (8.7–10.2)	8.1 (7.4–8.9)	−1.3 (−2.2 to −0.5)	<0.001	9.1 (8.53–9.76)	8.45 (7.67–8.65)	−1.01 (−2.28 to −0.025)	0.007	0.621
TAPSE	16 (12.2–20)	12 (10–15)	−3 (−8 to 0)	<0.001	16.5 (12.2–19.5)	12.5 (10–15.5)	−2.65 (−5.75 to −1)	0.030	0.578
TASV	10 (7–12)	8 (7–11)	−1 (−5 to 1)	0.001	10 (8.25–11)	8 (7–10.8)	−1 (−2.75 to 0)	0.087	0.834
sPAP	32.6 (24.7–41.2)	30 (26–33)	−3.89 (−11.7 to 7.01)	0.094	26.6 (19.6–37.5)	27.8 (25.2–33)	3.38 (−11.6 to 12)	0.766	0.383

Values are median (IQR). Δ indicates within-patient change (6 months − baseline). Within-sex *p*-values are from paired Wilcoxon signed-rank tests; between-sex *p*(Δ) is from Mann–Whitney *U* test. LVEDD, left ventricular end-diastolic diameter; LVSI, left ventricular sphericity index; PDI, right ventricular pressure–dimension index; sPAP, systolic pulmonary artery pressure; TASV, tricuspid annular systolic velocity; TAPSE, tricuspid annular plane systolic excursion.

#### 3.2.1 LVSI

In men, LVSI increased significantly from baseline to 6 months (median change ΔLVSI +0.045, within-group *p* = 0.001). In women, LVSI did not change significantly over the same period (median ΔLVSI −0.057, *p* = 0.766). The between-sex difference in ΔLVSI did not reach statistical significance (*p* = 0.098). Six-month LVSI values were similar between sexes (0.757 [0.650–0.862] vs. 0.708 [0.615–0.837], *p* = 0.455) (Table 2; Figure 2A).

#### 3.2.2 RV pressure–dimension index (PDI)

In men, PDI decreased markedly from baseline to 6 months (median change ΔPDI −1.475, *p* < 0.001). In women, the reduction was smaller and not statistically significant (median ΔPDI −0.922, *p* = 0.130). The between-sex difference in ΔPDI was not significant (*p* = 0.262). However, women had higher absolute PDI values at 6 months (2.01 [1.35–2.66] vs. 1.41 [0.90–2.50], *p* = 0.041); six-month PDI was missing in two patients (Table 2; Figure 2B). In contrast, women demonstrated a pattern of “geometric resistance” [defined descriptively as stable left ventricular sphericity index (ΔLVSI *p* = 0.766) and a non-significant change in RV pressure-dimension index (median ΔPDI −0.922, *p* = 0.130)], in contrast to adverse sphericalization and PDI decline observed in men.

Sex-stratified paired trajectories and summary statistics are shown in Figure 2, and the distribution of change scores (Δ = 6 months − baseline) is shown in Supplementary Figures S1A,B.

#### 3.2.3 Other echocardiographic remodeling parameters

Both sexes demonstrated effective LV unloading. Left ventricular end-diastolic diameter (LVEDD) decreased in men (median Δ −0.43 cm, *p* = 0.007) and women (median Δ −1.05 cm, *p* = 0.021), without a significant between-sex difference (*p* = 0.061). LV longitudinal diameter decreased significantly in both sexes (men median Δ −1.3 cm, *p* < 0.001; women median Δ −1.0 cm, *p* = 0.007). Regarding RV systolic function, TAPSE declined in both sexes (men median Δ −3.0 mm, *p* < 0.001; women median Δ −2.7 mm, *p* = 0.030). TASV declined significantly in men (median Δ −1.0 cm/s, *p* = 0.001) but showed a non-significant change in women (median Δ −1.0 cm/s, *p* = 0.087). Estimated sPAP showed no significant within-group change in either sex (Table 2; Figure 2).

### 3.3 Adjusted sex effects and sensitivity analysis

In ANCOVA-style models adjusting for baseline value, age, BMI, INTERMACS level, and device type, female sex was not independently associated with 6-month LVSI (*p* = 0.788) or 6-month PDI in robust mean-based models (*p* = 0.225; Table 3). However, in median regression analysis, female sex was associated with a significantly higher median 6-month PDI (adjusted difference +0.823, *p* = 0.031; Table 3). To examine the robustness of these findings, a prespecified 1:2 propensity score–matched sensitivity analysis (18 women matched to 36 men) was performed and achieved good covariate balance (absolute SMD < 0.10 for age, BMI, INTERMACS level, and device type; Supplementary Figure S2; Supplementary Table S1). In the matched cohort, the adjusted sex effect on 6-month PDI was attenuated and no longer statistically significant in median regression (adjusted median difference 0.828, 95% CI −0.150 to 1.806; *p* = 0.095; Supplementary Table S2, matched 1:2 row), suggesting that the observed differences were modest and may be influenced by baseline characteristics.

**Table 3:** Adjusted sex effect on 6-month values (female vs. male).

Cohort	Outcome	Model	Adjusted difference (female–male)	95% CI	*p*-value
Full cohort	6-month LVSI	Linear regression (robust SE)	−0.014	−0.120 to 0.091	0.788
Full cohort	6-month PDI	Linear regression (robust SE)	0.412	−0.258 to 1.082	0.225
Full cohort	6-month PDI	Quantile regression (median)	0.823	0.079 to 1.567	**0.031**
Matched cohort (1:2)	6-month LVSI	Linear regression (cluster-robust SE)	−0.060	−0.189 to 0.069	0.342
Matched cohort (1:2)	6-month PDI	Linear regression (cluster-robust SE)	0.276	−0.589 to 1.140	0.51
Matched cohort (1:2)	6-month PDI	Quantile regression (median)	0.828	−0.150 to 1.806	0.095

Models were adjusted for the baseline value of the outcome, age, body mass index, INTERMACS level, and device type. Adjusted differences are reported as female–male. Robust linear regression models report mean differences with robust standard errors; quantile regression reports the adjusted median difference. In the matched cohort, linear models used cluster-robust standard errors by matched set. CI, confidence interval; INTERMACS, Interagency Registry for Mechanically Assisted Circulatory Support; LVSI, left ventricular sphericity index; PDI, right ventricular pressure–dimension index; SE, standard error.

Bold value indicates statistical significance at *p* < 0.05.

### 3.4 Prognostic associations with mortality and RHF

#### 3.4.1 Survival by sex

**Figure 3:**
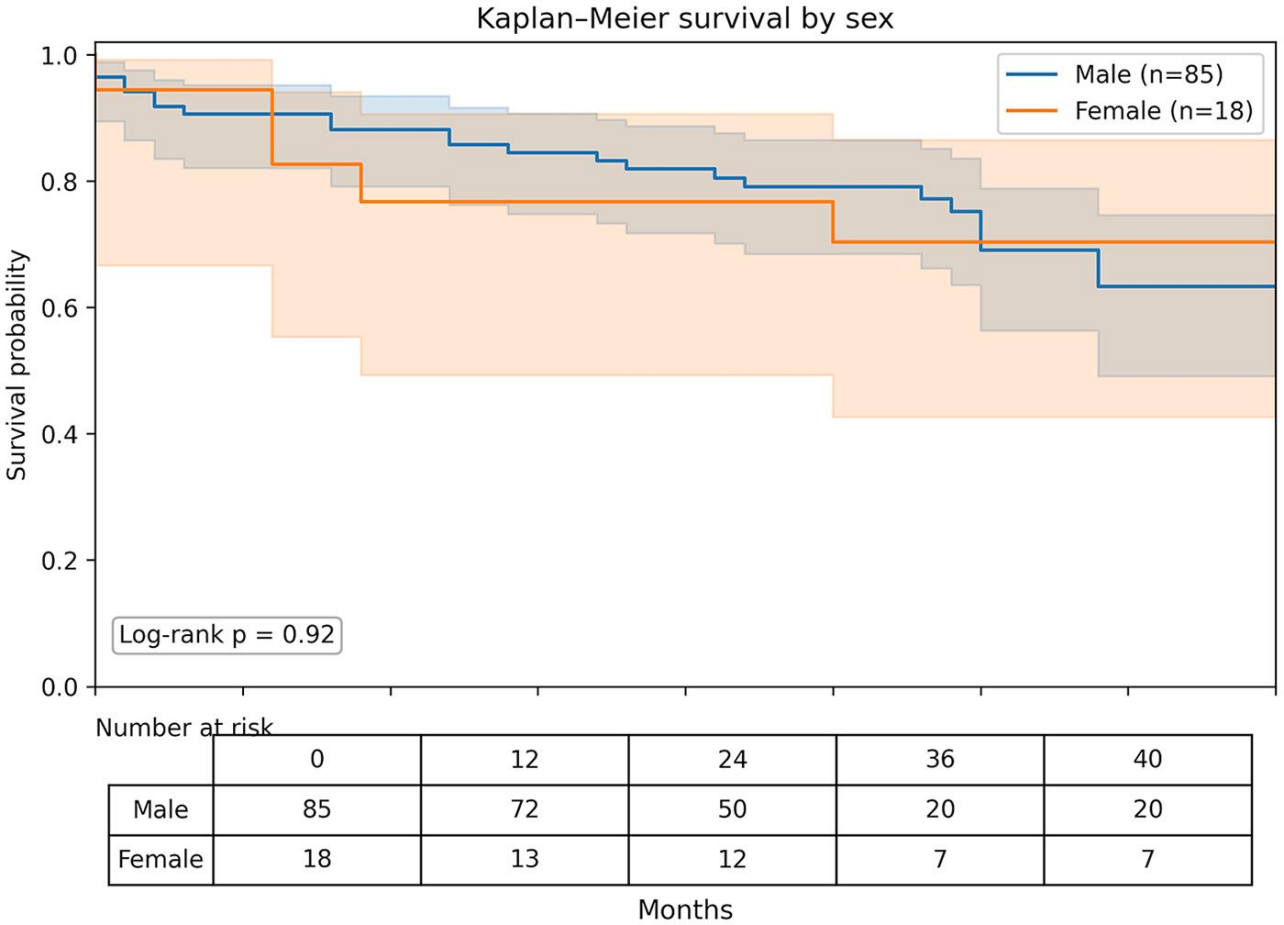
Kaplan–Meier survival curves by sex after LVAD implantation.

Kaplan–Meier curves showed no significant difference in survival between women and men (log-rank *p* = 0.920; Figure 3), consistent with univariable Cox regression (female sex: HR 0.95, 95% CI 0.36–2.49; *p* = 0.915; Table 4).

**Table 4:** Univariable Cox proportional hazards models for all-cause mortality (selected predictors).

Variable	HR (95% CI)	*p*
Baseline LVSI	1.43 (1.02–2.00)	0.038
Baseline PDI	1.16 (0.83–1.61)	0.388
GWI	0.13 (0.06–0.28)	<0.001
GCW	0.20 (0.10–0.37)	<0.001
GWE	0.30 (0.20–0.46)	<0.001
GWW	2.04 (1.46–2.84)	<0.001
LV-GLS	1.27 (0.89–1.82)	0.188
Female sex	0.95 (0.36–2.49)	0.915
Device (HM3 vs. HMII)	0.53 (0.25–1.13)	0.101

Values are hazard ratios (HR) with 95% confidence intervals (CI), reported per 1 standard deviation (SD) increase in the predictor. Two-sided *p*-values are shown. GWI, global work index; GCW, global constructive work; GWE, global work efficiency; GWW, global wasted work; LV-GLS, left ventricular global longitudinal strain; LVSI, Left ventricular sphericity index; PDI, right ventricular pressure–dimension index; HMII, HeartMate II; HM3, HeartMate 3.

#### 3.4.2 Prognostic associations with mortality

Higher baseline LVSI was associated with increased mortality risk in univariable analysis (HR 1.43 per SD, 95% CI 1.02–2.00; *p* = 0.038; Table 4) and remained significant after adjustment for sex, age, BMI, and device type (HR 1.47 per SD, 95% CI 1.03–2.10; *p* = 0.034; Table 5). Baseline myocardial work indices were strongly prognostic in univariable models (per SD; Table 4). Specifically, higher GWI (HR 0.13, 95% CI 0.06–0.28), GCW (HR 0.20, 95% CI 0.10–0.37), and GWE (HR 0.30, 95% CI 0.20–0.46) were protective against mortality, whereas higher GWW (HR 2.04, 95% CI 1.46–2.84) was associated with worse survival (all *p* < 0.001; Supplementary Figure S3; Supplementary Table S4).

**Table 5:** Adjusted Cox model including baseline LVSI.

Variable	HR (95% CI)	*p*
Baseline LVSI	1.47 (1.03–2.10)	0.034
Female sex	0.82 (0.31–2.20)	0.696
Age	0.77 (0.51–1.16)	0.206
BMI	0.73 (0.49–1.08)	0.117
Device (HM3 vs. HMII)	0.58 (0.27–1.24)	0.16

Model includes baseline LVSI (per SD) and is adjusted for sex, age (per SD), BMI (per SD), and device type (HM3 vs. HMII). LVSI, left ventricular sphericity index; BMI, body mass index; HMII, HeartMate II; HM3, HeartMate 3.

**Figure 4:**
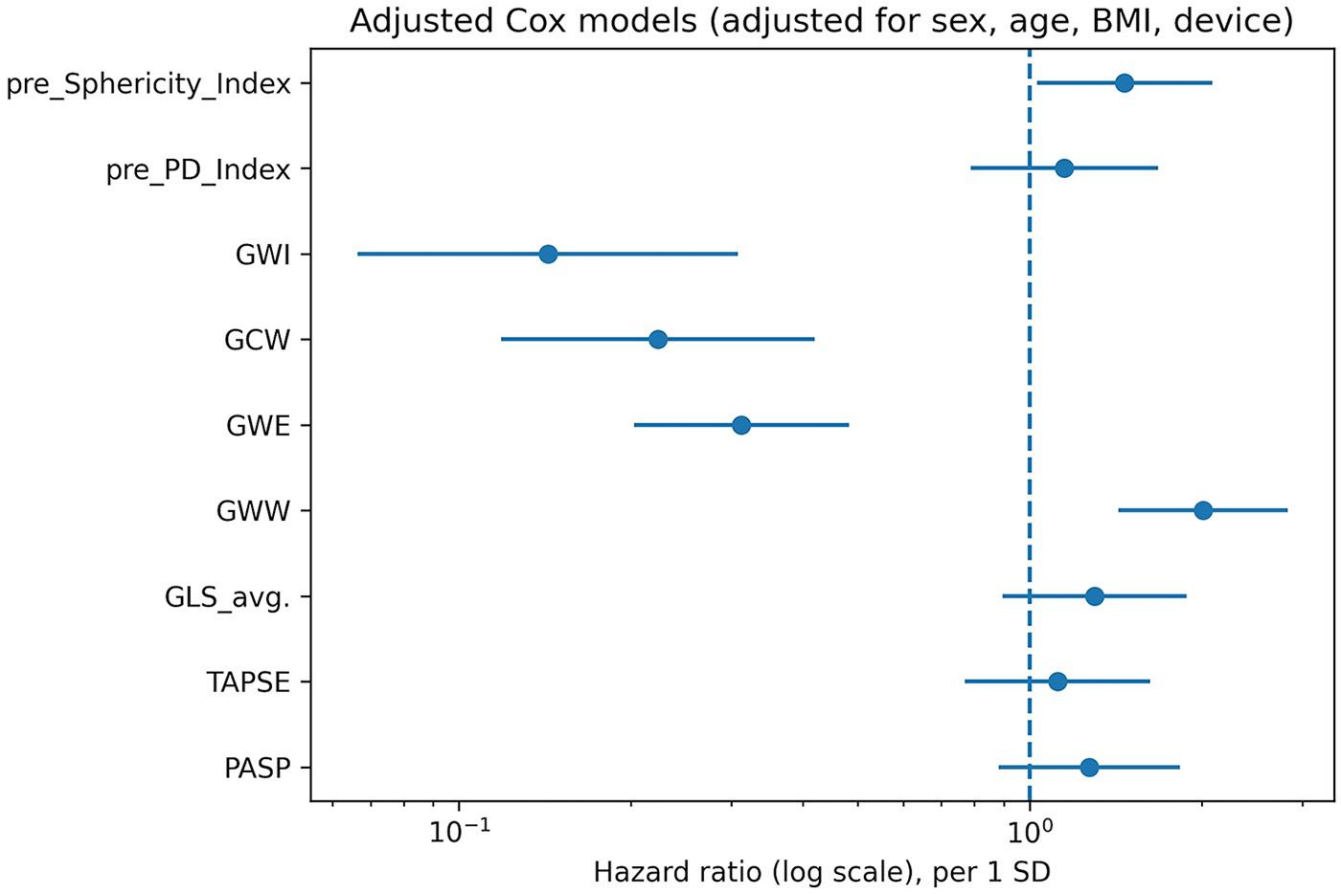
Adjusted associations of baseline LV geometry and myocardial work indices with all-cause mortality after LVAD implantation. Forest plot of hazard ratios (HR) from Cox proportional hazards models evaluating baseline LVSI (preoperative sphericity index), PDI (right ventricular pressure–dimension index), myocardial work indices (GWI, GCW, GWE, GWW), GLS, TAPSE, and sPAP in relation to mortality. HRs are shown per 1 standard deviation (SD) increase in each predictor (log-scale). Models were adjusted for sex, age, BMI, and device type (HM3 vs. HMII). Points indicate HRs and horizontal lines indicate 95% confidence intervals: the vertical dashed line marks HR = 1.0 (no association).

In the adjusted model, GWI remained independently associated with survival (HR 0.14 per SD, 95% CI 0.06–0.30; *p* < 0.001; Table 6). As illustrated in the adjusted Cox models (Figure 4), this independent prognostic signal extended to other myocardial work components: GCW and GWE remained associated with improved survival after multivariable adjustment, while GWW emerged as an independent predictor of mortality. Overall, pressure–strain-derived myocardial work markers showed stronger associations with mortality than conventional echocardiographic parameters included in the adjusted models (Figure 4).

**Table 6:** Adjusted Cox model including global work index (GWI).

Variable	HR (95% CI)	*p*
GWI	0.14 (0.06–0.30)	<0.001
Female sex	0.70 (0.26–1.86)	0.471
Age	0.84 (0.58–1.20)	0.335
BMI	0.93 (0.59–1.45)	0.745
Device (HM3 vs. HMII)	0.76 (0.35–1.63)	0.479

Model includes baseline GWI (per SD) and is adjusted for sex, age (per SD), BMI (per SD), and device type (HM3 vs. HMII). BMI, body mass index; GWI, global work index; HMII, HeartMate II; HM3, HeartMate 3.

#### 3.4.3 Postoperative right heart failure

In univariable logistic regression (per 1 SD increase), baseline TAPSE was strongly protective against postoperative RHF (OR 0.25, 95% CI 0.13–0.51; *p* < 0.001; Table 7). Higher baseline myocardial work indices were also protective, including GWI (OR 0.52, 95% CI 0.30–0.89; *p* = 0.017) and GWE (OR 0.55, 95% CI 0.34–0.88; *p* = 0.012; Table 7). In the multivariable logistic regression model including TAPSE and GWI and adjusted for sex, age, BMI, and device type, both TAPSE (OR 0.22, 95% CI 0.10–0.51; *p* < 0.001) and GWI (OR 0.41, 95% CI 0.20–0.88; *p* = 0.022) remained independently associated with RHF (Table 8).

**Table 7:** Univariable logistic regression models for postoperative right heart failure (RHF) (selected predictors).

Variable	OR (95% CI)	*p*
Baseline TAPSE	0.25 (0.13–0.51)	<0.001
GWI	0.52 (0.30–0.89)	0.017
GWE	0.55 (0.34–0.88)	0.012
GWW	1.34 (0.88–2.04)	0.178
Baseline LVSI	1.21 (0.78–1.86)	0.392
Baseline PDI	1.25 (0.82–1.89)	0.305
Female sex	0.93 (0.27–3.14)	0.905
Device (HM3 vs. HMII)	1.85 (0.71–4.82)	0.206

Values are odds ratios (OR) with 95% confidence intervals (CI), reported per 1-SD increase in the predictor. Two-sided *p* values are shown. CI, confidence interval; GWI, global work index; GWE, global work efficiency; GWW, global wasted work; HMII, HeartMate II; HM3, HeartMate 3; LVSI, left ventricular sphericity index; OR, odds ratio; PDI, right ventricular pressure–dimension index; RHF, right heart failure; SD, standard deviation; TAPSE, tricuspid annular plane systolic excursion.

**Table 8:** Multivariable logistic regression model for postoperative right heart failure (RHF) including baseline TAPSE and GWI.

Variable	OR (95% CI)	*p*
Baseline TAPSE	0.22 (0.10–0.51)	<0.001
GWI	0.41 (0.20–0.88)	0.022
Female sex	0.57 (0.19–1.74)	0.326
Age	0.93 (0.53–1.63)	0.806
BMI	0.91 (0.57–1.46)	0.691
Device (HM3 vs. HMII)	2.40 (0.75–7.73)	0.142

Model includes baseline TAPSE and GWI (each per SD) and is adjusted for sex, age (per SD), BMI (per SD), and device type (HM3 vs. HMII). Values are odds ratios (OR) with 95% confidence intervals (CI). Two-sided *p* values are shown. BMI, body mass index; CI, confidence interval; GWI, global work index; HMII, HeartMate II; HM3, HeartMate 3; OR, odds ratio; RHF, right heart failure; SD, standard deviation; TAPSE, tricuspid annular plane systolic excursion.

### 3.5 Exploratory outcome sensitivity analysis in the broader LVAD cohort

As an exploratory sensitivity analysis addressing potential selection bias, we repeated sex-specific outcome analyses in the broader available LVAD cohort, including patients initially excluded from the primary echocardiographic analysis. Because excluded patients lacked analyzable paired echocardiography and/or valid myocardial work data, they could not be incorporated into the remodeling models. However, survival time, vital status, and postoperative RHF status were available for 231 of the 244 screened patients. In this broader cohort, female sex was not associated with all-cause mortality in Kaplan–Meier analysis (log-rank *p* = 0.499), univariable Cox regression (HR 1.13, 95% CI 0.79–1.61; *p* = 0.511), or adjusted Cox regression including age, BMI, INTERMACS level, and device type (HR 1.02, 95% CI 0.70–1.48; *p* = 0.917). Similarly, female sex was not associated with postoperative RHF (28.9% vs. 24.5%; Fisher's exact *p* = 0.524; adjusted OR 1.31, 95% CI 0.69–2.48; *p* = 0.413; Supplementary Table S3). These findings suggest that the sex-specific clinical outcome results were not solely driven by selection into the echocardiographic analysis cohort.

## 4 Discussion

This exploratory single-center study provides a sex-specific analysis of early biventricular remodeling and prognostic echocardiographic markers after durable LVAD implantation. The main hypothesis-generating findings were: (1) men and women showed different early remodeling trajectories during the first 6 months of LVAD support, with men showing increased LVSI and declining RV PDI, whereas women showed relative geometric stability; (2) higher preoperative LVSI was associated with long-term mortality; and (3) non-invasive myocardial work, particularly GWI, was associated with both postoperative RHF and all-cause mortality.

### 4.1 Sex-specific patterns of geometric remodeling

Sex differences in heart failure outcomes and LVAD survival have been widely observed, yet the structural mechanisms driving these disparities remain incompletely characterized (6, 7). Our study adds a mechanical perspective to this literature. We observed that while LVAD support effectively unloaded the left ventricle in both sexes (evidenced by significant reductions in LVEDD), the *shape* of the ventricle evolved differently. Male patients exhibited “adverse geometric remodeling”—becoming significantly more spherical over time—while female patients demonstrated “geometric resistance” (as defined in Section 3.2), maintaining their preoperative shape with a non-significant trend toward normalization.

This finding contrasts with prior work by Kenigsberg et al. (20), who reported that women experienced greater early reverse remodeling after LVAD implantation, including greater reductions in LV dimensions and greater improvement in LVEF compared with men. However, our data suggest that volume reduction does not always equate to geometric normalization. The tendency for women to retain a more elliptical geometry may reflect fundamental biological differences in myocardial stiffness and fibrosis patterns. As noted by Regitz-Zagrosek et al. (21), female hearts in heart failure with reduced ejection fraction (HFrEF) often display distinct extracellular matrix remodeling, characterized by less replacement fibrosis and higher passive stiffness compared to male hearts (21). This “stiffer” phenotype may physically resist the spherical distortion that often accompanies prolonged mechanical unloading and septal shifting in men.

This preservation of LV geometry in women was accompanied by a more favorable adaptation of the right ventricle. While the PDI—a marker of RV adaptation to load—decreased in the overall cohort, women retained significantly higher absolute PDI values at 6 months compared to men (*p* = 0.041). A higher PDI implies better maintenance of RV pressure generation capacity relative to dilation (9, 10). These findings may, in part, reflect sex-associated differences in cardiac reverse remodeling during mechanical unloading with LVAD support, consistent with prior reports showing more favorable longitudinal changes in cardiac structure and function among women after contemporary LVAD implantation (20).

### 4.2 Registry context and interpretation

These findings should also be interpreted in the context of larger LVAD registry data. In an INTERMACS-based analysis, women represented approximately one-fifth of LVAD recipients and had higher adjusted risks of mortality and post-implant adverse events, including bleeding, stroke, rehospitalization, and pump thrombosis or device malfunction (6). In contrast, contemporary European PCHF-VAD registry data reported no statistically significant adjusted survival difference between women and men, despite sex-related differences in baseline profile and LVAD utilization (7). Similarly, in a large contemporary HeartMate 3 analysis, women and men had comparable 2-year survival, although women experienced higher rates of stroke, bleeding, and major infection after implantation (22). These discrepancies may reflect differences in device era, patient selection, body size, adverse-event profiles, transplant probability, and competing risks. Our cohort was not powered to resolve registry-level discrepancies in clinical outcomes between sexes. Rather, the present study provides exploratory mechanistic echocardiographic observations that may help generate hypotheses for future multicenter or registry-based imaging studies.

### 4.3 Prognostic value of LV geometry

Beyond sex differences, our data reinforce the critical importance of ventricular geometry in risk stratification. We found that a more spherical LV at baseline (higher LVSI) was independently associated with increased mortality (HR 1.47 per SD). This aligns with the concept that sphericalization represents a maladaptive, end-stage phenotype associated with increased wall stress and energetic inefficiency, as described by Konstam et al. (8). Recent reviews emphasize that the transition from an elliptical to a spherical shape alters fiber orientation and impairs torsional mechanics, thereby reducing pump efficiency even when ejection fraction remains unchanged (23). Our findings suggest that patients who present with a “rounder” ventricle are at higher risk of adverse long-term outcomes, regardless of the degree of unloading achieved.

### 4.4 Myocardial work and right heart failure

A novel aspect of this study is the evaluation of non-invasive myocardial work indices in the specific context of LVAD candidates. Unlike LVEF or GLS, which are strictly load-dependent, MW indices incorporate afterload (via cuff pressure), offering a more robust estimation of myocardial metabolic demand and contractility (12, 13).

We found that preoperative GWI was independently associated with postoperative RHF (OR 0.41 per SD), providing prognostic value alongside the established marker TAPSE. This is consistent with emerging data from Landra et al. (24), who recently demonstrated that right ventricular myocardial work indices are superior to conventional echocardiographic parameters in predicting early RHF after LVAD implantation (24). Our findings extend this by showing that *left* ventricular GWI also holds predictive power, highlighting the importance of interventricular dependence: patients with higher “constructive” LV work reserves likely possess a more robust interventricular septum, which is the primary driver of RV function (25). Furthermore, lower preoperative GWI and GWE were associated with all-cause mortality. These findings support further prospective evaluation of pressure–strain-derived myocardial work indices as complementary markers in pre-implant echocardiographic risk assessment, consistent with contemporary multimodality imaging recommendations and prior myocardial work studies in LVAD candidates (5, 15).

### 4.5 Clinical implications

Our findings may have practical implications for the echocardiographic evaluation of advanced heart failure patients considered for durable mechanical circulatory support, but they require validation in larger cohorts. First, the observed sex-specific remodeling trajectories suggest that postoperative echocardiographic surveillance may benefit from a sex-aware interpretation; the lack of geometric normalization in women should not automatically be viewed as insufficient unloading, but may represent a distinct phenotype of relative geometric stability.

Second, the prognostic association of LVSI supports further evaluation of LVSI as a simple adjunctive marker during preoperative screening. However, the present study was not designed to derive or validate a clinical LVSI threshold. Therefore, LVSI values approaching or exceeding 0.75 should be regarded as hypothesis-generating markers of advanced adverse geometry rather than validated decision cut-offs. Prospective studies with formal threshold analyses and external validation are required before LVSI can be used to guide clinical decision-making. Finally, myocardial work indices may provide complementary information during pre-LVAD implantation evaluation and should be evaluated prospectively before routine clinical implementation.

### 4.6 Limitations

This study has limitations inherent to its retrospective, single-center design. Although the total cohort size (*n* = 103) is comparable to other mechanistic echocardiographic studies, the number of female patients was relatively small (*n* = 18), reflecting the known epidemiology of the LVAD population. This limited our statistical power to detect smaller between-sex differences in mortality or secondary remodeling endpoints. With only 18 female patients, the study had limited statistical power to detect smaller between-sex differences in the primary remodeling endpoint (between-sex ΔLVSI *p* = 0.098). A *post-hoc* power calculation for the observed difference in ΔLVSI, assuming *α* = 0.05 and the observed variability, indicated approximately 45% power; the minimum detectable effect size for 80% power was approximately 0.12 LVSI units. Consequently, the non-significant between-sex result should be interpreted as inconclusive rather than as definitive evidence of equivalence.

The 6-month remodeling analysis is subject to survivor bias because 20 patients (14 deaths and 6 heart transplantations) did not reach the 6-month echocardiographic assessment (see Figure 1). Attrition rates due to death or transplantation before 6 months were comparable between sexes (men: 14/177 [7.9%]; women: 6/67 [9.0%]). The requirement for sinus rhythm and absence of relevant extrasystoles may have introduced selection bias, as atrial fibrillation and rhythm irregularities are frequent in advanced heart failure and LVAD candidates. Although necessary for reliable speckle-tracking and myocardial work analysis, this criterion may limit generalizability to patients with persistent atrial fibrillation or non-sinus rhythm. Furthermore, the primary remodeling analysis was restricted to a fixed 6-month echocardiographic time point, precluding assessment of longer-term sex-specific remodeling trajectories. Finally, while non-invasive estimates of PDI and MW have been validated (9, 15), they are not a substitute for invasive hemodynamic monitoring.

## 5 Conclusion

In this exploratory single-center cohort, women demonstrated a remodeling pattern consistent with preserved LV geometry and PDI after LVAD implantation; however, these sex-specific findings should be interpreted cautiously given the small number of female patients and attenuation after propensity-score matching. Independent of sex, baseline LV sphericity and non-invasive myocardial work showed prognostic associations with mortality and right heart failure, supporting further validation in larger multicenter cohorts and future risk-stratification studies.

## Data Availability

The raw data supporting the conclusions of this article will be made available by the authors, without undue reservation.
