# Supplementary material for: Sex-specific echocardiographic remodeling and prognostic value of left ventricular sphericity, pressure–dimension index, and myocardial work after durable LVAD implantation
Source: Front Cardiovasc Med. 2026 Jun 23;13:1815815. doi: 10.3389/fcvm.2026.1815815 (PMC13337415; doi:10.3389/fcvm.2026.1815815)
Supplement: Supplementary file 1 [file Datasheet1.docx]

Supplementary Material

# Supplementary Figures and Tables

## Supplementary Figures

**Supplementary Figure S1. Sex-specific change in LVSI and PDI from baseline to 6 months after LVAD implantation.**

**
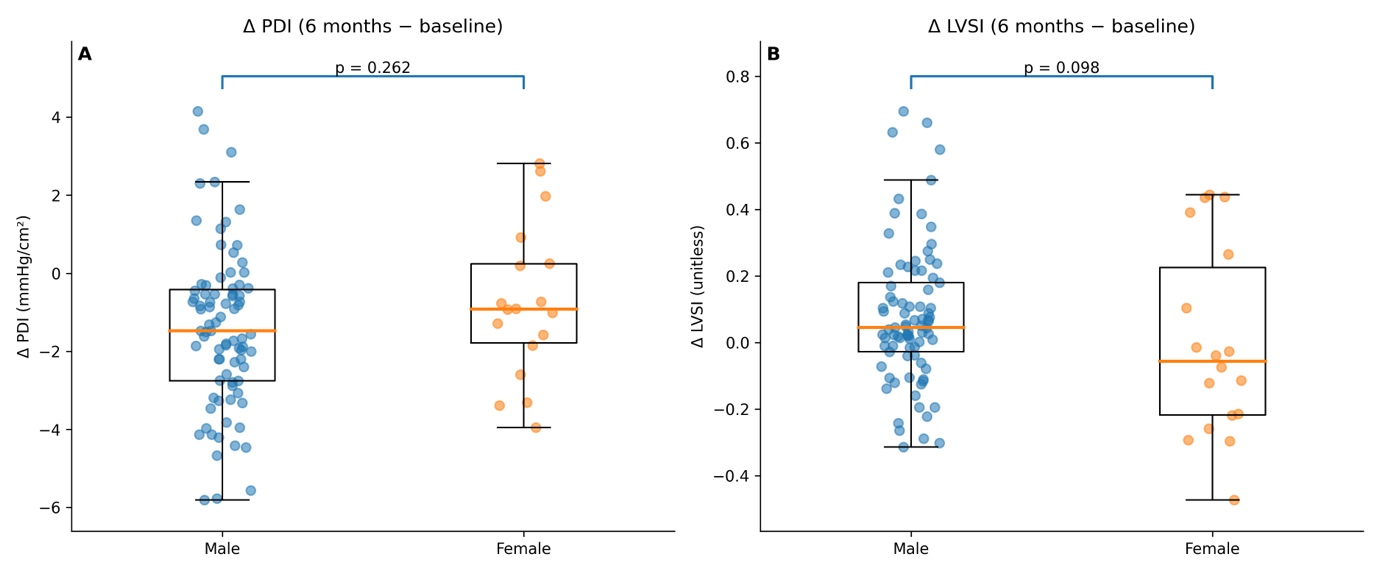
**

*(A) Change in pressure–dimension index (PDI) and (B) change in left ventricular sphericity index (LVSI), calculated as Δ = 6 months − baseline. Boxplots display the median and interquartile range (IQR; 25th–75th percentile); whiskers indicate values within 1.5×IQR, and points represent individual patients. Brackets show the between-sex p-value for the difference in Δ (Mann–Whitney U test).*

**Supplementary Figure S2. Covariate balance before and after 1:2 propensity score matching.**


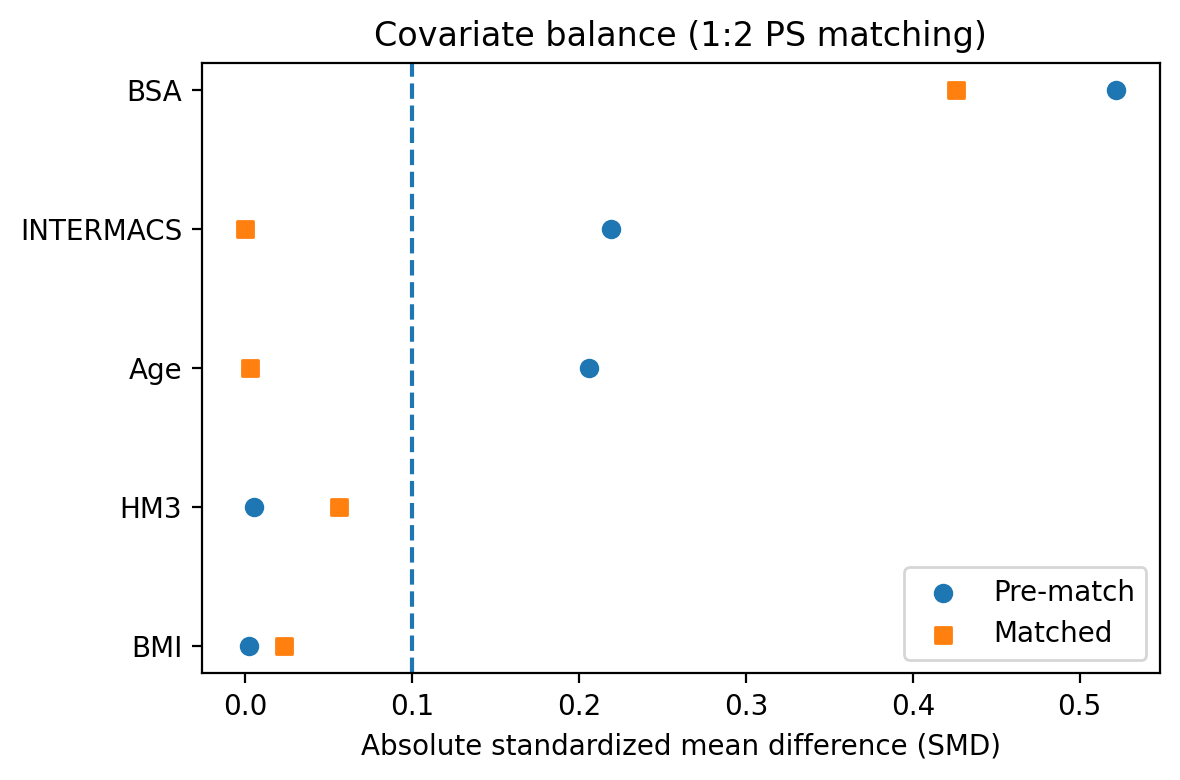

*Love plot showing absolute standardized mean differences (SMD) for matching covariates (****age, BMI, INTERMACS level, device type****) and* ***BSA*** *before and after matching (18 women matched to 36 men). The dashed vertical line at* ***SMD=0.10*** *indicates the prespecified threshold for acceptable balance. Matching improved balance for the prespecified covariates, while* ***BSA remained imbalanced****, reflecting sex-related differences in body size not included in the matching model.*

**Supplementary Figure S3. Univariable prognostic associations of baseline echocardiographic markers with all-cause mortality.**

**
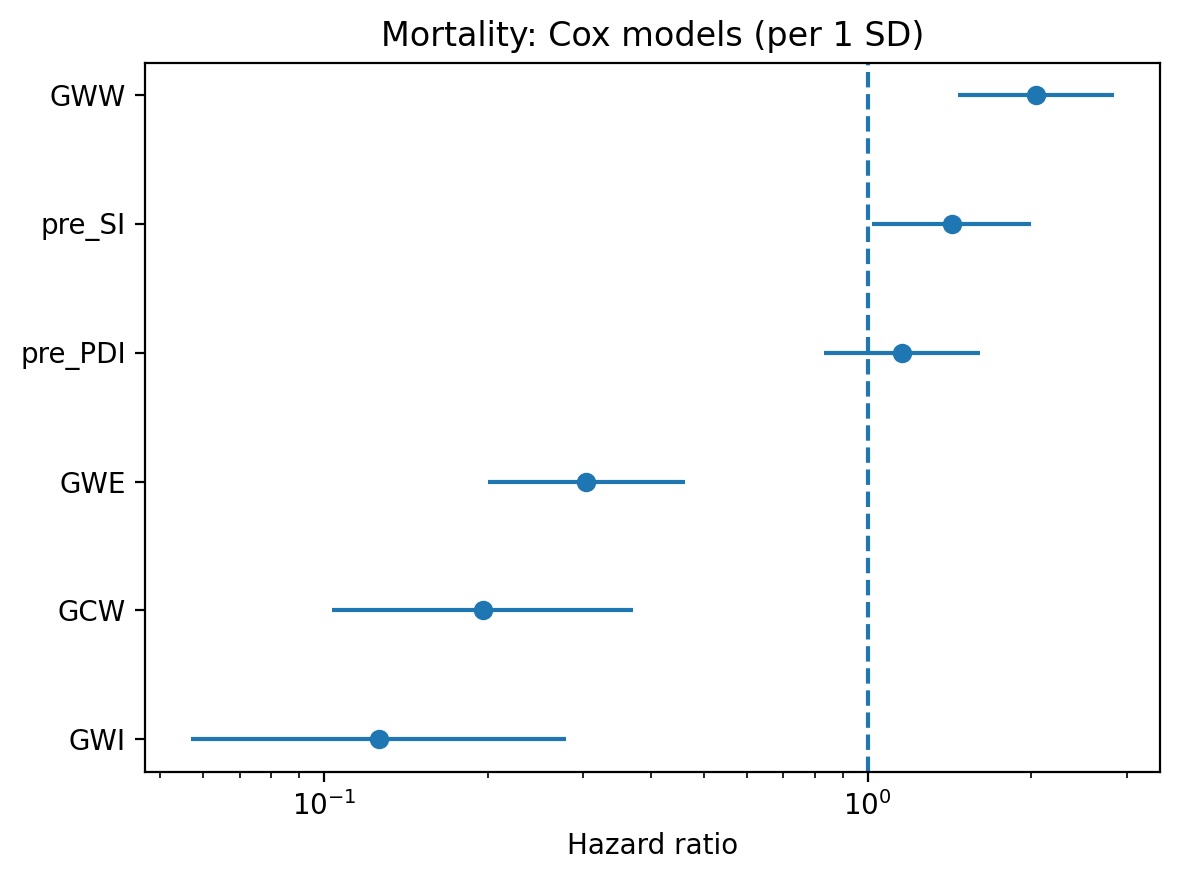
**

*Forest plot displaying hazard ratios (HR) from univariable Cox proportional hazards models for the prediction of all-cause mortality. The markers evaluated include: GWW: Global wasted work; pre_SI: Preoperative left ventricular sphericity index (LVSI); pre_PDI: Preoperative right ventricular pressure-dimension index; GWE: Global work efficiency; GCW: Global constructive work; GWI: Global work index Hazard ratios are reported per 1 standard deviation (SD) increase in each continuous predictor. Points represent the hazard ratio, and horizontal lines indicate the 95% confidence intervals. The vertical dashed line at HR = 1.0indicates the null hypothesis (no association). Note that GWI, GCW, and GWE are strongly protective (lower HR), while higher LVSI and GWW are associated with increased mortality risk.*

## Supplementary Tables

**Supplementary Table S1. Covariate balance before and after 1:2 propensity score matching (Female vs Male).**

| Covariate | \|SMD\| pre | \|SMD\| matched |
| --- | --- | --- |
| BMI | 0.00 | 0.02 |
| HM3 | 0.01 | 0.06 |
| Age | 0.21 | 0.00 |
| INTERMACS | 0.22 | 0.00 |

*Absolute standardized mean differences (|SMD|) are shown for key matching variables before matching (“pre”) and after matching (“matched”). Adequate covariate balance was defined a priori as |SMD| <0.10. Abbreviations: BMI, body mass index; HM3, HeartMate 3 device; INTERMACS, Interagency Registry for Mechanically Assisted Circulatory Support.*

**Supplementary Table S2. Adjusted association of sex (Female vs Male) with 6-month echocardiographic measures in the full cohort and in the 1:2 propensity score–matched cohort.**

| Cohort | Outcome | Model | Sex effect (Female vs Male) | 95% CI | p |
| --- | --- | --- | --- | --- | --- |
| Full cohort | 6-month sphericity index (SI) | OLS (HC3) | -0.014 | -0.120 to 0.091 | 0.788 |
| Full cohort | 6-month pressure–diameter index (PDI) | OLS (HC3) | 0.412 | -0.258 to 1.082 | 0.225 |
| Full cohort | 6-month pressure–diameter index (PDI) | Median regression | 0.823 | 0.079 to 1.567 | 0.031 |
| Matched 1:2 | 6-month sphericity index (SI) | OLS (cluster by set) | -0.060 | -0.189 to 0.069 | 0.342 |
| Matched 1:2 | 6-month pressure–diameter index (PDI) | OLS (cluster by set) | 0.276 | -0.589 to 1.140 | 0.510 |
| Matched 1:2 | 6-month pressure–diameter index (PDI) | Median regression | 0.828 | -0.150 to 1.806 | 0.095 |

Values are regression coefficients representing the adjusted difference in the outcome for females compared with males. For OLS models, coefficients represent adjusted mean differences; for median regression, coefficients represent adjusted median differences. Full-cohort models used heteroscedasticity-consistent (HC3) robust standard errors. Matched-cohort models accounted for matching by using standard errors clustered by matched set. Abbreviations: CI, confidence interval; OLS, ordinary least squares; PDI, pressure–diameter index; SI, sphericity index.

**Supplementary Table S3. Exploratory outcome sensitivity analysis in the broader available LVAD cohort.**

| **Outcome / analysis** | **Men** | **Women** | **Effect estimate** | **p-value** |
| --- | --- | --- | --- | --- |
| **Cohort size** | 155 | 76 | — | — |
| **All-cause mortality** | 83/155 (53.5%) | 48/76 (63.2%) | — | — |
| Kaplan–Meier survival comparison | — | — | Log-rank test | 0.499 |
| Univariable Cox regression for all-cause mortality | — | — | HR 1.13 (95% CI 0.79–1.61) | 0.511 |
| Adjusted Cox regression for all-cause mortality | — | — | HR 1.02 (95% CI 0.70–1.48) | 0.917 |
| **Postoperative right heart failure** | 38/155 (24.5%) | 22/76 (28.9%) | — | — |
| Fisher’s exact test for right heart failure | — | — | — | 0.524 |
| Univariable logistic regression for right heart failure | — | — | OR 1.25 (95% CI 0.68–2.32) | 0.471 |
| Adjusted logistic regression for right heart failure | — | — | OR 1.31 (95% CI 0.69–2.48) | 0.413 |

The broader available cohort included 231 LVAD recipients with available survival time, vital status, and postoperative right heart failure status, including patients initially excluded from the primary echocardiographic analysis. Cox regression models assessed all-cause mortality; logistic regression models assessed postoperative right heart failure. Adjusted models included sex, age, body mass index, INTERMACS level, and device type. Effect estimates are shown for female versus male sex. Values are shown as n/N (%) unless otherwise indicated.

**Abbreviations:**
BMI, body mass index; CI, confidence interval; HR, hazard ratio; INTERMACS, Interagency Registry for Mechanically Assisted Circulatory Support; LVAD, left ventricular assist device; OR, odds ratio.
